# Supplementary material for: A cross-sectional study evaluating tick-borne encephalitis vaccine uptake and timeliness among adults in Switzerland
Source: PLoS One. 2021 Dec 14;16(12):e0247216. doi: 10.1371/journal.pone.0247216 (PMC8670666; doi:10.1371/journal.pone.0247216)
Supplement: S1 Table — (DOCX) [file pone.0247216.s001.docx]

**S1 Table. Swiss Large Geographic Regions and Integrated Cantons.**

| **Large Region** | **Integrated Cantons** |
| --- | --- |
| Lake Geneva | Geneva, Vaud, Valais |
| Ticino | Ticino |
| Midland Switzerland | Bern, Solothurn, Freiburg, Neuchâtel, Jura |
| Northwest Switzerland | Basel-Stadt, Basel-Landschaft, Aargau |
| Central Switzerland | Uri, Schwyz, Obwalden, Nidwalden, Lucerne, Zug |
| East Switzerland | St. Gallen, Thurgau, Appenzell Innerrhoden, Appenzell Ausserrhoden, Glarus, Schaffhausen, Graubunden |
| Zurich | Zurich |

Adapted from: https://www.bfs.admin.ch/bfs/en/home/statistics/regional-statistics.html
