# Supplementary material for: A cross-sectional study evaluating tick-borne encephalitis vaccine uptake and timeliness among adults in Switzerland
Source: PLoS One. 2021 Dec 14;16(12):e0247216. doi: 10.1371/journal.pone.0247216 (PMC8670666; doi:10.1371/journal.pone.0247216)
Supplement: S2 Table — (DOCX) [file pone.0247216.s002.docx]

**S2 Table. Determination of Study Sample Size.**

| **Formula** | ***n*=((*z*^2^*p*(1-*p*))\|*e*^2^ + ((*z*^2^*p*(1-*p*))\|*N*))\|*R*** |
| --- | --- |
| Precision (*e*) | 0.05 |
| Prevalence (*p*) | 0.5 (Conservative Estimate) |
| Confidence Interval (*z*) | 1.96 |
| Population Size^a^ (*N*) | 6,570,644 |
| Predicted Response Rate (*R*) | 0.3^b^ |

^a^Adult population of Switzerland, aged 18-79, in 2018 from the Swiss Federal Statistical Office (https://www.bfs.admin.ch/bfs/en/home.html)

^b^Based on results from the Swiss National Vaccination Coverage Survey [3].
