# Supplementary material for: A cross-sectional study evaluating tick-borne encephalitis vaccine uptake and timeliness among adults in Switzerland
Source: PLoS One. 2021 Dec 14;16(12):e0247216. doi: 10.1371/journal.pone.0247216 (PMC8670666; doi:10.1371/journal.pone.0247216)
Supplement: S3 Table — (DOCX) [file pone.0247216.s003.docx]

**S3 Table. Timing of TBE Booster Vaccine Uptake Prior to and After 2006.**

|  | **Days between Doses 3-4 pre-2006** | **Days between Doses 3-4 post-2006** | **Days between Doses 4-5 pre-2006** | **Days between Doses 4-5 post-2006** |
| --- | --- | --- | --- | --- |
| Number of values | 139 | 344 | 62 | 94 |
| Mean | 1338 (3.7 years) | 3135 (8.6 years) | 1309 (3.6 years) | 2412 (6.6 years) |
| Lower 95% CI of mean | 1235 | 2926 | 1109 | 2135 |
| Upper 95% CI of mean | 1467 | 3257 | 1424 | 2802 |
| Median | 1136 (3.1 years) | 3528 (9.7 years) | 1128 (3.1 years) | 2493 (6.8 years) |
| Lower 95% CI of median | 1119 | 3410 | 1100 | 1859 |
| Upper 95% CI of median | 1198 | 3583 | 1167 | 3604 |
| Range | 6-4375 | 9-9868 | 30-3633 | 19-5931 |
